# Supplementary material for: Multi‐dataset identification of innovative feature genes and molecular mechanisms in keratoconus
Source: J Cell Mol Med. 2024 Sep 19;28(18):e70079. doi: 10.1111/jcmm.70079 (PMC11412914; doi:10.1111/jcmm.70079)
Supplement: Supplementary file 2 — Material S2. [file JCMM-28-e70079-s002.zip › 7.GSEA analysis/1.ARL11/KEGG_PPAR_SIGNALING_PATHWAY.html]

Details for gene set KEGG\_PPAR\_SIGNALING\_PATHWAY[GSEA]

|  || Dataset | ARL11.group.cls#h\_versus\_l |
| Phenotype | group.cls#h\_versus\_l |
| Upregulated in class | h |
| GeneSet | KEGG\_PPAR\_SIGNALING\_PATHWAY |
| Enrichment Score (ES) | 0.23852311 |
| Normalized Enrichment Score (NES) | 0.78329223 |
| Nominal p-value | 0.82107353 |
| FDR q-value | 1.0 |
| FWER p-Value | 1.0 |
Table: GSEA Results Summary

  

Fig 1: Enrichment plot: KEGG\_PPAR\_SIGNALING\_PATHWAY      
 Profile of the Running ES Score & Positions of GeneSet Members on the Rank Ordered List

  

| SYMBOL | TITLE | RANK IN GENE LIST | RANK METRIC SCORE | RUNNING ES | CORE ENRICHMENT || 1 | ACADL | na | 70 | 0.704 | 0.0744 | Yes |
| 2 | FABP7 | na | 764 | 0.420 | 0.1001 | Yes |
| 3 | CYP8B1 | na | 1368 | 0.351 | 0.1208 | Yes |
| 4 | ADIPOQ | na | 1547 | 0.333 | 0.1519 | Yes |
| 5 | GK2 | na | 1619 | 0.326 | 0.1853 | Yes |
| 6 | CYP7A1 | na | 2190 | 0.283 | 0.1995 | Yes |
| 7 | AQP7 | na | 2205 | 0.282 | 0.2297 | Yes |
| 8 | SLC27A2 | na | 2836 | 0.248 | 0.2385 | Yes |
| 9 | PLTP | na | 4210 | 0.193 | 0.2199 | No |
| 10 | FABP1 | na | 4567 | 0.180 | 0.2292 | No |
| 11 | APOA1 | na | 5619 | 0.148 | 0.2149 | No |
| 12 | PCK1 | na | 5840 | 0.141 | 0.2239 | No |
| 13 | CYP4A11 | na | 6286 | 0.129 | 0.2251 | No |
| 14 | HMGCS2 | na | 6474 | 0.125 | 0.2333 | No |
| 15 | FADS2 | na | 7752 | 0.097 | 0.2070 | No |
| 16 | CPT2 | na | 7848 | 0.095 | 0.2146 | No |
| 17 | ACOX1 | na | 9236 | 0.071 | 0.1823 | No |
| 18 | PLIN1 | na | 9314 | 0.070 | 0.1877 | No |
| 19 | CPT1A | na | 9745 | 0.064 | 0.1822 | No |
| 20 | SORBS1 | na | 9757 | 0.063 | 0.1888 | No |
| 21 | RXRA | na | 9938 | 0.061 | 0.1902 | No |
| 22 | PDPK1 | na | 10673 | 0.052 | 0.1746 | No |
| 23 | ACADM | na | 11858 | 0.038 | 0.1446 | No |
| 24 | SCD5 | na | 11918 | 0.037 | 0.1469 | No |
| 25 | ACSL1 | na | 12605 | 0.030 | 0.1304 | No |
| 26 | SCD | na | 12612 | 0.030 | 0.1335 | No |
| 27 | SLC27A5 | na | 12940 | 0.028 | 0.1271 | No |
| 28 | PPARA | na | 13194 | 0.025 | 0.1225 | No |
| 29 | APOC3 | na | 13214 | 0.025 | 0.1247 | No |
| 30 | FABP5 | na | 13398 | 0.024 | 0.1220 | No |
| 31 | APOA2 | na | 14248 | 0.017 | 0.0993 | No |
| 32 | ACSL5 | na | 14433 | 0.016 | 0.0957 | No |
| 33 | ACSL4 | na | 15066 | 0.011 | 0.0786 | No |
| 34 | FABP6 | na | 15164 | 0.010 | 0.0769 | No |
| 35 | SLC27A4 | na | 15361 | 0.008 | 0.0722 | No |
| 36 | ACAA1 | na | 15725 | 0.006 | 0.0623 | No |
| 37 | SLC27A6 | na | 16425 | 0.001 | 0.0422 | No |
| 38 | SCP2 | na | 17655 | -0.008 | 0.0076 | No |
| 39 | DBI | na | 18162 | -0.011 | -0.0057 | No |
| 40 | ACOX3 | na | 18292 | -0.012 | -0.0081 | No |
| 41 | CYP27A1 | na | 19243 | -0.019 | -0.0335 | No |
| 42 | NR1H3 | na | 20994 | -0.031 | -0.0806 | No |
| 43 | UBC | na | 21024 | -0.032 | -0.0780 | No |
| 44 | ILK | na | 21090 | -0.032 | -0.0764 | No |
| 45 | UCP1 | na | 21225 | -0.033 | -0.0767 | No |
| 46 | RXRB | na | 22268 | -0.043 | -0.1021 | No |
| 47 | PPARD | na | 23551 | -0.056 | -0.1330 | No |
| 48 | ACSL3 | na | 24299 | -0.066 | -0.1473 | No |
| 49 | SLC27A1 | na | 24351 | -0.067 | -0.1415 | No |
| 50 | EHHADH | na | 24876 | -0.074 | -0.1486 | No |
| 51 | APOA5 | na | 25560 | -0.084 | -0.1592 | No |
| 52 | FABP3 | na | 26046 | -0.091 | -0.1633 | No |
| 53 | CPT1B | na | 26241 | -0.094 | -0.1587 | No |
| 54 | CD36 | na | 26649 | -0.102 | -0.1594 | No |
| 55 | CYP4A22 | na | 27781 | -0.125 | -0.1784 | No |
| 56 | ACSL6 | na | 28020 | -0.131 | -0.1710 | No |
| 57 | ACOX2 | na | 28878 | -0.154 | -0.1790 | No |
| 58 | PPARG | na | 28947 | -0.155 | -0.1641 | No |
| 59 | ANGPTL4 | na | 29257 | -0.165 | -0.1552 | No |
| 60 | CPT1C | na | 29364 | -0.168 | -0.1400 | No |
| 61 | GK | na | 30263 | -0.196 | -0.1446 | No |
| 62 | PCK2 | na | 30472 | -0.203 | -0.1286 | No |
| 63 | ME1 | na | 31396 | -0.235 | -0.1296 | No |
| 64 | FABP4 | na | 31465 | -0.238 | -0.1058 | No |
| 65 | OLR1 | na | 32902 | -0.315 | -0.1130 | No |
| 66 | RXRG | na | 33017 | -0.323 | -0.0812 | No |
| 67 | MMP1 | na | 33608 | -0.370 | -0.0580 | No |
| 68 | LPL | na | 33967 | -0.415 | -0.0233 | No |
| 69 | FABP2 | na | 33976 | -0.416 | 0.0217 | No |
Table: GSEA details [plain text format]

  

Fig 2: KEGG\_PPAR\_SIGNALING\_PATHWAY      
 Blue-Pink O' Gram in the Space of the Analyzed GeneSet

  

Fig 3: KEGG\_PPAR\_SIGNALING\_PATHWAY: Random ES distribution      
 Gene set null distribution of ES for **KEGG\_PPAR\_SIGNALING\_PATHWAY**
